# Compatibility effects with destination and origin of motion

**DOI:** 10.1371/journal.pone.0281829

**Published:** 2023-02-17

**Authors:** Elisa Scerrati, Roberto Nicoletti, Sandro Rubichi, Claudia Scorolli, Luisa Lugli

**Affiliations:** 1 Department of Biomedical, Metabolic and Neural Sciences, University of Modena e Reggio Emilia, Modena, Italy; 2 Department of Philosophy and Communication, University of Bologna, Bologna, Italy; Universite de Poitiers, FRANCE

## Abstract

Previous studies highlighted spatial compatibility effects other than those strictly arising from stimulus-response locations. In particular, the so-called Destination Compatibility (DC) effect refers to faster responses for dynamic (i.e., moving) stimuli the end point of which is spatially compatible with the response key. Four experiments examined whether the DC effect also occurs with static visual stimuli symbolically representing either motion destination alone (Experiment 1a), or both motion origin and destination (Experiments 1b, 2a, and 2b). Overall, our results are consistent in showing a DC effect; most importantly, the present findings reveal a predominance of the effect of destination of motion over that of origin, even when both the starting and ending positions of the stimulus are symbolically represented and participants are instructed to respond according to motion origin. This finding suggests that the DC effect is independent from other stimulus-response compatibility (SRC) effects.

## Introduction

Motion conveys information that might be critical for human beings. For instance, in sports, such as a typical volleyball match, one might focus visual attention on the opposing players’ motions as well as on the ball’s motion after an opposing player hits it in order to determine the planning and execution of the resulting reception action. However, does motion need to be unfolding to be helpful to plan and execute a response? And does motion destination outweigh motion origin or viceversa? The present study seeks responses to these questions by using a stimulus-response compatibility (SRC) paradigm with a choice reaction task.

The spatial stimulus-response compatibility effect refers to faster and more accurate responses when a certain spatial arrangement of the stimulus is compatible with the spatial arrangement of the response [1, 2; see also 3, 3]. For example, people are faster and more accurate at responding to a sensory stimulation appearing on the right of their body’s midline with a response-key positioned on the right rather than on the left [5]. This effect is found even when the spatial arrangement of the stimulus is irrelevant to the task. For example, in the Simon effect [6, 7; see also 3, 4] discriminating a non-spatial characteristic of the stimulus, such as color or shape, produces faster and more accurate responses if a right-positioned stimulus requires a response with a right rather than a left key.

Research highlighted spatial compatibility effects other than those strictly arising from the overlap of stimulus-response locations. Hommel [8] investigated the spatial compatibility effect concerning stimulus location and the location of the effect the subject aims to produce (i.e., spatial stimulus-effect compatibility). In his study it has been demonstrated a response facilitation when there is the intention to produce an effect (e.g., turn on a light) on a side opposite to that of responding and the stimulus and effect sides overlap. More specifically, participants responded faster to the pitch of target tones through left- and right-hand keypresses when these tones were delivered through a loudspeaker located on the same side as the light lighting up as a result of the response.

Even more relevant to the aim of the present study, Ansorge [9] investigated the spatial compatibility relationship concerning the intention to produce a left-right motion and the response location (i.e., spatial intention-response compatibility), whereas Kunde [10] examined the spatial compatibility relationship concerning the effect resulting from a certain action and the response location (i.e., response-effect compatibility). Specifically, Ansorge [9] presented participants with a central stimulus, either the letter H or T, and asked them to move it toward the right or the left on the screen by pressing one of the two mouse keys. The author found that responses were faster when people had to press a key located in the same direction as the intended motion. On the other hand, Kunde [10] asked participants to discriminate the color of a centrally presented circular dot by pressing one of four horizontally aligned keys (one for each of four possible colors). As a result of the key press, one of four horizontally aligned boxes presented on the screen above the keys became white. Results showed faster responses when the position of the box turning white and the response key were compatible (i.e., one above the other) rather than incompatible (i.e., one adjacent to the other). Therefore, both the intention to produce a certain motion and the consequences or effects of certain actions spark off spatial compatibility effects. In a similar vein, Scerrati, D’Ascenzo, Lugli, Iani, Rubichi, and Nicoletti [11] showed that the spatial compatibility between action-effects and response is able to counteract the spatial compatibility between object-manipulation and response. More specifically, the compatibility effect between handle and response did not emerge if the effect of the action was expected to occur on the horizontal axis, that is contralateral to the object’s grasping handle. However, the compatibility effect did emerge if the effect was expected on the vertical axis. This result indicates a crucial role of the functional component of graspable objects in the occurrence of compatibility effects between object-manipulation and response.

Importantly, all these findings provide support to the *common coding hypothesis of intention and action* [12, 13], and the *theory of event coding* [TEC: 14] that in line with the *ideomotor principle* [15; see also 16 for an earlier formulation of the same principle] assume that we represent our intentions and actions in terms of their anticipated sensory consequences [see also 17–19]. Accordingly, a common representational code is shared by perceived stimuli and generated actions. Therefore, effects can act as stimuli that, although being irrelevant to the task or situation, are being processed and are able to influence responses [see 20 for a review].

Further evidence shows an effect of the destination of apparent motion on response. Michaels [21] demonstrated that responding to stimuli that move left and right on the screen is easier when the response is performed with a joystick spatially compatible with the direction of motion, i.e., a destination compatibility (DC) effect. Proctor, Van Zandt, Lu, and Weeks [22] extended this finding by showing the emergence of the DC effect with keypress responses, regardless of whether motion was conveyed by a stimulus that expanded, contracted, or changed position on screen. Importantly, they found a similar effect by using static arrow stimuli to represent destinations. To illustrate, in their Experiment 5 rather than signalling motion destination with motion on screen of one of two squares, two static squares appeared on the computer screen, and after an interval, an arrow appeared inside one square. The arrow pointed at either the ipsilateral or contralateral response location. It is well known that the arrows indicate a certain location in much the same way as does the direction of motion since compatibility effects have been shown with centrally located arrows pointing left and right [23–25]. Indeed, their results showed a DC effect for static arrows stimuli.

Furthermore, Tagliabue, Falciati, Umiltà, and Massaccesi [26] investigated destinations, i.e., the effects that the action produces, in an ecological setting. Indeed, the authors used moving stimuli in the context of a simulated, familiar, everyday behavior, namely driving a car. Participants had to push a joystick either toward a lit-up traffic light or in the opposite direction depending on whether the light was green or red. In the Coordinated Information condition, the car turned in the same direction in which the joystick was moved (i.e., it turned right if people pushed their joystick to the right and it turned left if people pushed their joystick to the left). In the Uncoordinated Information condition, the car turned in the opposite direction than where the joystick was moved (i.e., it turned right if people pushed their joystick to the left and it turned left if people pushed their joystick to the right). Consistent with the destination-response compatibility hypothesis, they found a facilitation in making a car turning left or right by moving a joystick in the direction spatially compatible with the car’s motion direction, that is, in the condition with coordinated information, indicating facilitated responding when there is correspondence between the direction in which the hand moves and the destination to be reached. More recently, Classen and Kibele [27]

More recently, Classen and Kibele [27] investigated the visual perception of approaching or departing movements of inanimate objects (i.e., spheres) and real people in a three-dimensional space. The response buttons were near and far from the observer, and the task-relevant information was color change in the video. Although the direction of movement was irrelevant information for the purposes of the task, the authors found a DC effect with both types of stimuli. That is, participants responded faster when the direction of movement of the dynamic stimulus matched the spatial location of the required response. Importantly, in the discussion the authors suggest the possible presence of a weighting mechanism of static and dynamic spatial information with stronger weighting for the former type of information. Specifically, they suggest that "static position information of the stimulus is more similar to the spatial features of the demanded response since pressing a key on a fixed location does not involve substantial dynamics" [p. 141].

The studies by Tagliabue et al. [26] and that by Classen and Kibele [27] converge in demonstrating a DC effect in the case of dynamic stimuli. Both studies are also characterized by an effort to pursue new possibilities for the expansion of experimental research on attentional processes—overcoming the limitations of traditional research, which is characterized by the artificiality of the procedures and scenarios proposed. In our work we maintain a commitment to lead experimental research toward greater ecological validity and to explore phenomena that have an impact on our everyday behaviors (in our case, the field investigated is that of sports as we focus on a typical volleyball match). With respect to these two studies, however, we go further: indeed, our goal is to examine whether the DC effect persists even in the case of static visual stimuli that symbolically represent the destination of movement.

This consistent body of evidence suggests that motion perception affects the planning, selection and execution of responses. However, previous studies focused on motion either as the consequence of participants’ actions [e.g., 9, 26] or as evoked by dynamic (i.e., moving) stimuli displayed on screen [e.g., 21, 27; but see 22 for a different approach with arrow stimuli]. Furthermore, as far as we know, no previous study compared the effects of motion origin (i.e., starting position of the stimulus) and destination (i.e., ending position of the stimulus) on response with static visual stimuli, which are not arrows. Therefore, three overlooked issues deserve a systematic investigation: 1) whether a DC effect is observed when motion destination is independent from participants’ intentions and actions contrary to when the participant’s response makes stimuli changing on screen [e.g., 9, 26]; 2) whether a DC effect can also be elicited by a static stimulus, which, however, is not an arrow as in [22] but a simple line suggesting the trajectory of an object’s motion; 3) what happens when the DC effect is pitted against the effect of motion origin. To address these issues, we conducted four experiments. Experiment 1a was devoted to test whether a symbolically represented object destination that appears on screen independently from the participant response action, and which is not an arrow is able to influence response. Experiment 2a was conceived to compare the effect of the destination of motion with that of the origin of motion to control for potential effects of spatial information. Experiments 1b and 2b aimed at further testing the results obtained from Experiments 1a and 2a, respectively and were conducted online [e.g., 28–30].

## Experiment 1a

In Experiment 1a, participants were presented with static pictures of a volleyball court where an attack action was unfolding. The ball trajectory was represented by a static diagonal line that appeared below a central volleyball ball and could be oriented to the left or right of the court. Depending on the assigned group, participants had to respond to the predicted ball destination (Destination-Instructions group) or to the predicted position of the opposing player’s hand that hit the ball (Hand-Instructions group) by pressing either a spatially-compatible (compatible session) or a spatially-incompatible (incompatible session) key on the keyboard. In the Destination-Instructions group we expect to find a spatial DC effect (i.e., faster responses for spatially compatible than incompatible destination and response) even if participants’ response does not trigger a motion on screen, and no dynamic stimulus was used. Conversely, in the Hand-Instructions group we expect to observe a spatial compatibility effect concerning the predicted position of the opposing player’s hand and the response, due a shift of participants visual attention to the opposite side than the ball destination as required by instructions.

### Method

#### Participants

We calculated the sample size required to achieve 60% power [31–33] to detect a significant main effect of Spatial compatibility (compatible, incompatible) with the G*power 3.1 [34] software. With a medium effect size f = 0.25 [35], the power calculation recommended a total sample size of at least 22 participants. The same calculation was used in the following experiments where the same predictions were tested.

Twenty-four students from the University of Bologna (18 women; mean age = 20.4 years, SD = 1.4) took part in this experiment voluntarily. One participant was excluded from the sample because he took several breaks during the practice and interrupted the experiment after a while. All participants had normal or corrected-to-normal vision and two of them reported themselves as being left-handed. They were not volleyball players or volleyball trainers and they were naive as to the purpose of the experiment.

This and the following experiments were conducted in accordance with the ethical standards laid down in the Declaration of Helsinki and fulfilled the ethical standard procedure recommended by the Italian Association of Psychology (AIP). All procedures were approved by the ethics committee of the University of Bologna (Approval number 322426). All participants gave their written informed consent to participate.

#### Material and apparatus

In order to represent a volleyball court, two versions of the same image were created by using Microsoft Power Point. Both consisted of a vertically aligned rectangle (height: 12 cm, width: 10 cm; 11° x 9.5° of visual angle) split in two halves by a straight horizontal line. At the center of the rectangle, a graphically stylized volleyball ball (height: 0.67 cm, width: 0.67 cm; 0.64° x 0.64° of visual angle) was shown. A 0.4 cm height line (0.38° of visual angle), with 45-degree tilt, appeared below the ball, on the left or on the right side indicating the trajectory of the ball.

Stimuli were presented on a Dell 22-inch (56 cm) video monitor (refresh rate: 60 Hz; resolution: 1280 × 800 pixels) on a white background. The viewing distance was 60 cm. Stimuli presentation and responses collection were controlled by E-Prime Professional v2.0 software (Psychology Software Tools, Inc., Sharpsburg, PA).

#### Procedure

The experiment was conducted individually in a quiet room where the light was dimmed. Participants completed a compatible and an incompatible session, each encompassing 2 blocks of 120 trials. In each block, images appeared in a randomized order. Each session was preceded by a practice phase of 24 trials identical to the experimental ones. Participants were informed about the beginning and the end of the practice phase and that it could help them familiarize with the task. The whole experiment lasted approximately 20 minutes.

The instructions described the rectangle as a volleyball court where an attack action was unfolding, the horizontal line as the net that divided the court, the tilted line as the trajectory of the ball after an alleged hand, located opposite to the ball trajectory, hit it.

Participants were randomly assigned to one of two groups: the Destination-Instructions group (N = 12) and the Hand-Instructions group (N = 11). Participants in the Destination-Instructions group were asked to respond with a response-key on the left (i.e., key 3 from the top keyboard numbers) if the ball was directed to the left, and with a response-key on the right (i.e., key 9 from the top keyboard numbers) if the ball was directed to the right in the compatible session. Conversely, in the incompatible session, they were asked to respond with a response-key on the left (key 3) if the ball was directed to the right, and with a response-key on the right (key 9) if the ball was directed to the left.

Participants in the Hand-Instructions group were instructed to respond with a response-key on the left (key 3) if the alleged hand hitting the ball was on the left, and with a response-key on the right (key 9) if the alleged hand was on the right in the compatible session. Conversely, in the incompatible session, they were instructed to respond with a response-key on the left (key 3) if the alleged hand hitting the ball was on the right, and with a response-key on the right (key 9) if the alleged hand was on the left. For both Instruction groups (destination, hand), the order of sessions was counterbalanced across participants.

Each trial began with a fixation cross displayed in the centre of the screen for 600 ms, then the stimulus appeared at the screen centre for 600 ms. Participants had to respond within the 600 ms frame otherwise their response was not recorded, that trial was considered timed-out and the next trial appeared on the screen. After each response a feedback (i.e., “correct”, “incorrect” or “too late”) was provided for 1000 ms.

### Results

Practice trials, incorrect responses (3.78% of trials) and omissions (1.49% of trials) were excluded from the analysis on the response times (RTs). RTs higher and lower than 2 standard deviations from the participant mean were also excluded (4.69% of trials). No participant was excluded from the analyses as the single participant’s error rate was generally low (less than 25%) in all four experiments, therefore we had no reason to suspect people misconceived the task or performed it inattentively.

A mixed ANOVA with the within-subject factor *Spatial compatibility* (compatible vs. incompatible) and the between-subjects factor *Instructions group* (destination vs. hand) was conducted on RTs. To note, for this and the following experiments spatial compatibility was computed between the ball destination and the response location for the Destination-Instructions group, whereas it was computed between the predicted location of the opponent player’s hand and the response location for the Hand-Instructions group.

Crucially, there was a significant two-way interaction between *Spatial compatibility* and *Instructions group*, *F*(1,21) = 109.64, Mse = 14514.54, *p <* .001, *η*_*p*_^2^ = .839. All other effects failed to reach significance, F_s_ < 1. Planned comparisons aimed at comparing spatially-compatible and incompatible responses in the two instructions groups (destination, hand) revealed that for the Destination-Instructions group, compatible responses, i.e. responses given using a response-key ipsilateral to the ball destination (M: 323 ms SE: 5.9) were faster than incompatible responses (M: 361 ms, SE: 9.1), i.e. responses given using a response-key contralateral to the ball destination, resulting in a 38 ms significant DC effect, *t*(11) = 7.17, *p* < .001. On the contrary, for the Hand-Instructions group, compatible responses, i.e. responses given using a response-key ipsilateral to the opposing player’s hand (M: 352 ms SE: 8.1) were slower than incompatible responses (M: 320 ms SE: 8.9) i.e. responses given using a response-key contralateral to the opposing player’s hand, resulting in a -32 ms significant reversed hand-response compatibility effect, *t*(10) = 8.16, *p* < .001.

A mixed ANOVA with the same factors was conducted on the percentage errors (PEs). Crucially, there was a significant two-way interaction between *Spatial compatibility* and *Instructions group*, *F*(1,21) = 11.06, Mse = 84.88, *p =* .003, *η*_*p*_^2^ = .345. All other effects failed to reach significance, F_s_ < 1. Planned comparisons aimed at comparing spatially-compatible and incompatible responses in the two instructions groups (destination, hand) revealed that for the Destination-Instructions group, compatible responses, i.e. responses given using a response-key ipsilateral to the ball destination (M: 2.5% SE: 0.6) tended to be more accurate than incompatible responses (M: 4.7%, SE: 1.5), i.e. responses given using a response-key contralateral to the ball destination. This difference, however, failed to reach statistical significance, *t*(11) = 1.80, *p* = .099. On the contrary, for the Hand-Instructions group, compatible responses, i.e. responses given using a response-key ipsilateral to the opposing player’s hand (M: 5.5%, SE: 0.9) were less accurate than incompatible responses (M: 2.3%, SE: 0.7) i.e. responses given using a response-key contralateral to the opposing player’s hand, resulting in a -3.2% significant reversed hand-response compatibility effect, *t*(10) = 3.10, *p* = .011.

### Discussion

In the Destination-Instructions group, results showed a spatial DC effect between the symbolically represented ball trajectory, which indicates the destination of the ball, and the response position. It is worth noting that this effect was obtained despite motion was neither the consequence of a participant’s response action [see, for example, 9, 26 where participants’ responses made stimuli moving on screen], nor conveyed by dynamic stimuli [see, for example, 21, 27 where moving stimuli were used]. This result suggests that the effects of an object supposed motion, that is, of an object motion never displayed on screen but the trajectory of which is symbolically represented on the monitor, can act as stimuli and influence responses, strengthening the hypothesis that we represent action in terms of the effects it produces [e.g., 12, 13].

Surprisingly, in the Hand-Instructions group responses were faster and more accurate for the hand-response spatially incompatible trials than for the hand-response spatially compatible trials, leading to a reversed hand-response compatibility effect. That is, a spatial DC effect was observed for this group too. This result seems to suggest that participants in the Hand-Instructions group ignored the task-relevant spatial information concerning the alleged hand hitting the ball. That is, despite Hand-Instructions focused participants’ attention on the side opposite to that of the ball destination, their attention was anchored to the line symbolically representing the trajectory of the ball, that is, to the side where they anticipated the ball would end up. Thus, motion destination seems to be preferentially coded even when people are explicitly required to attend to motion origin (i.e., the alleged hand hitting the ball).

However, this result might be due to the fact that in Experiment 1 the ball trajectory indicating motion destination was the only available spatial feature participants could rely on. To investigate this possibility, we conducted Experiment 1b, where the line indicating the ball trajectory was made symmetrical and superimposed on the ball.

## Experiment 1b

Experiment 1a obtained a DC effect with asymmetrical stimuli, that is, stimuli where only the line indicating the ball trajectory was visible with both instructions types: those emphasizing motion destination and those emphasizing motion origin. Experiment 1b aims at testing whether a DC effect occurs using symmetrical stimuli. To this end, we revised our stimuli such that the line indicating the ball trajectory, rather than appearing on the left or on the right below the volleyball ball, is superimposed on it and comes out of the same length upward and downward the ball. Ball destination was indicated by the dash. Experiment 1b was a web-based Experiment due to the intervening pandemic circumstances.

### Method

#### Participants

Twenty-four students from the University of Modena and Reggio Emilia (17 women; mean age = 22.1 years, SD = 6.2) participated in Experiment 1b. One of them reported himself as being left-handed. They were selected with the same criteria adopted for Experiment 1a.

#### Material and apparatus

**Fig 1:**
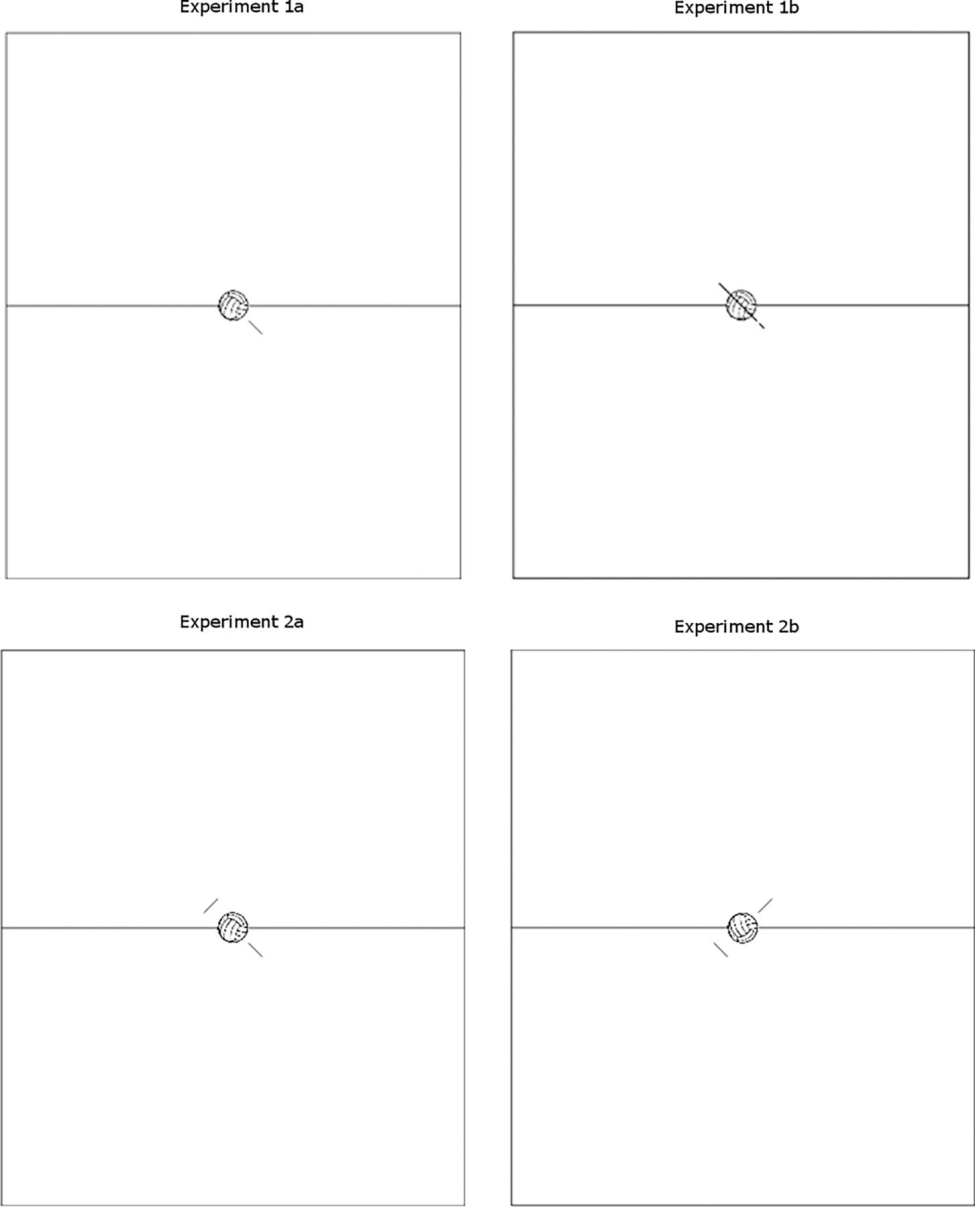
A schematic representation of the stimuli used across the four experiments (Top-left Panel = Experiment 1a; Top-right Panel = Experiment 1b; Bottom-left Panel = Experiment 2a; Bottom-right Panel = Experiment 2b). Note that only the condition with the right trajectory of the ball is reported. Elements in the present figure are not drawn to scale.

The stimuli were the same as in Experiment 1a except that one unique line indicating both motion origin and destination now appeared centrally, superimposed on the volleyball ball. A 1.2 cm height line (1.14° of visual angle), with 45-degree tilt, appeared on the ball from left to right or from right to left, indicating both the hitting hand (top) and the trajectory of the ball (bottom; see Fig 1, top-right Panel, for a schematic representation of the stimulus).

We used the Gorilla Experiment Builder (www.gorilla.sc) to create and host our web-based experiment [37, 38]. Automated procedures ensured that participants were all using a desktop computer and automatically rejected participants who took more than 2 hours to complete the task. To minimize potential distractions, participants were invited to carry out the experiment in a quiet place and to avoid the manipulation of any object throughout the task. In addition, before starting participants were asked to close background apps, software and all browser windows except for that of the experiment.

Responses were emitted by pressing the “e” (left) and “o” (right) keys of a QWERTY keyboard without the numeric pad, and the “y” (left) and “p” (right) keys of a QWERTY keyboard with the numeric pad.

#### Design and procedure

The design and procedure were the same as in Experiment 1a.

### Results

Practice trials, incorrect responses (5.63% of trials) and omissions (2.99% of trials) were excluded from the analysis on the response times (RTs). RTs higher and lower than 2 standard deviations from the participant mean were also excluded (4.73% of trials).

As in Experiment 1a, a mixed ANOVA with *Spatial compatibility* (compatible vs. incompatible) and *Instructions group* (destination vs. hand) was conducted on RTs. The main effect of *Spatial compatibility* was significant, *F*(1,22) = 6.01, Mse = 1.60, *p =* .023, *η*_*p*_^2^ = .215, indicating that compatible responses, i.e. responses given using a response-key ipsilateral to either the ball destination (in the Destination-Instructions group) or the opposing player’s hand (in the Hand-Instruction group) were faster (M: 392 ms SE: 6.4) than incompatible responses (M: 401 ms, SE: 6.0), i.e. responses given using a response-key contralateral to either the ball destination (in the Destination-Instructions group) or the opposing player’s hand (in the Hand-Instruction group). All other effects failed to reach significance, F_s_ < 1.

A mixed ANOVA with the same factors was conducted on the percentage errors (PEs). The main effect of *Spatial compatibility* was significant, *F*(1,22) = 8.72, Mse = .362, *p =* .007, *η*_*p*_^2^ = .284, indicating that compatible responses, i.e. responses given using a response-key ipsilateral to either the ball destination (in the Destination-Instructions group) or the opposing player’s hand (in the Hand-Instruction group) were more accurate (M: 4.3%, SE: 0.8) than incompatible responses (M: 6.8%, SE: 1.4), i.e. responses given using a response-key contralateral to either the ball destination (in the Destination-Instructions group) or the opposing player’s hand (in the Hand-Instruction group). All other effects failed to reach significance, F_s_ < 1. Despite the interaction between Spatial compatibility and Instructions group failed to reach significance (p = .920, p = .839 for RTs and PEs, respectively), for the sake of completeness and to comply with our hypotheses aimed at testing the robustness of the DC effect when pitted against the effect of motion origin, planned comparisons were conducted on the magnitude of the Spatial-compatibility effects (mean response time/percentage errors on incompatible trials minus mean response time/percentage errors on compatible trials), separately for each Instruction group for both dependent variables (RTs and PEs). RTs showed a significant DC effect of 9 ms in the Destination-instruction group, t(11) = 2.35, p = .038, and a non-significant hand-response compatibility effect of 9 ms in the Hand-instruction group, t(11) = 1.39, p = .189. PEs showed a significant DC effect of 2.7% in the Destination-instruction group, t(11) = 2.69, p = .021, and a non-significant hand-response compatibility effect of 2.3% in the Hand-instruction group, t(11) = 1.69, p = .118. Therefore, these additional exploratory t-tests suggest there was a DC effect for the Destination instruction group only, but since the interaction with Instructions group failed to show significance we should take the absence of a hand-response compatibility effect in the Hand-instruction group as only weak evidence (though, see the Alternative analyses in the S1 File section).

### Discussion

Results from Experiment 1b showed a main effect of Spatial compatibility.

Therefore, Experiment 1b seems to indicate that the stimulus arrangement may have a somewhat impact on the relevance of the ball destination when participants are instructed to respond to the position of the hitting hand since, unlike in Experiment 1a, a DC effect was not observed in the Hand-Instructions group with a symmetrical stimulus. It is worth noting that in Experiment 1b the stimulus arrangement did not separate motion origin from motion destination potentially causing ambiguity of spatial information. The following experiment aims at settling this issue.

## Experiment 2a

Experiment 1a demonstrated a spatial DC effect even when the instructions required participants to focus attention on the opposite side than the ball destination (Hand-Instructions group). In contrast, Experiment 1b showed a main effect of Spatial compatibility suggesting the stimulus arrangement may drive a origin-to-response compatibility effect when the instructions focus participants’ attention on motion origin and the stimulus is symmetrical. However, these results might be the consequence of an uneven availability of spatial information (as in Experiment 1a) or of a stimulus arrangement that did not separate motion origin from motion destination information (as in Experiment 1b). Therefore, in Experiment 2a we revised our stimuli such that both the ball trajectory (i.e., motion destination) and the opposing player’s hand (i.e., motion origin) were shown but they were indicated by separate lines. We expect to find an effect of the ball destination in the Destination-Instructions group and an effect of the hitting hand in the Hand-Instructions group.

### Method

#### Participants

Thirty-two students from the University of Bologna (21 women; mean age = 20.9 years, SD = 2.0) took part in Experiment 2a. One of them reported himself as being left-handed. They were selected with the same criteria adopted for the previous experiments.

#### Material and apparatus

The stimuli were the same as in Experiment 1a except that two lines of identical length now appeared, one below and one above the volleyball ball. A 0.4 cm height line (0.38° of visual angle), with 45-degree tilt, appeared below the ball, on the left or on the right side, indicating the trajectory of the ball; moreover a 0.4 cm width line (0.38° of visual angle), with 45 degree tilt, appeared above the ball, on the right or on the left side, indicating the hitting hand (see Fig 1 bottom-left Panel). The apparatus was identical to that in Experiment 1a.

#### Design and procedure

The design and procedure were the same as in Experiment 1a.

### Results

Practice trials, incorrect responses (3.62% of trials) and omissions (1.71% of trials) were excluded from the analysis on the response times (RTs). RTs higher and lower than 2 standard deviations from the participant mean were also excluded (5.02% of trials).

As in previous experiments, a mixed ANOVA with *Spatial compatibility* (compatible vs. incompatible) and *Instructions group* (destination vs. hand) was conducted on RTs. Crucially, there was a significant two-way interaction between *Spatial compatibility* and *Instructions group*, *F*(1,30) = 7.58, Mse = 2282.76, *p =* .010, *η*_*p*_^2^ = .202. All other effects failed to reach significance, F_s_ < 2.10, p_s_ > .157. Planned comparisons aimed at comparing spatially-compatible and incompatible responses in the two instructions groups (destination, hand) revealed that for the Destination-Instructions group, compatible responses, i.e. responses given using a response-key ipsilateral to the ball destination (M: 345 ms SE: 7.7) were faster than incompatible responses (M: 364 ms, SE: 10.0), i.e. responses given using a response-key contralateral to the ball destination, resulting in a 18 ms significant DC effect, *t*(15) = 2.68, *p* = .017. On the contrary, for the Hand-Instructions group, compatible responses, i.e. responses given using a response-key ipsilateral to the opposing player’s hand (M: 352 ms SE: 6.1) were slightly slower than incompatible responses (M: 346 ms SE: 7.5) i.e. responses given using a response-key contralateral to the opposing player’s hand, resulting in a -6 ms non-significant reversed hand-response compatibility effect, *t*(15) = 1.04, *p* = .311.

An ANOVA with the same factors was conducted on the percentage errors. The interaction between *Spatial compatibility* and *Instructions group* failed to reach significance, *F* = 3.36, p = .077. All other effects were non significant, *F*_*s*_ < 1.

### Discussion

Results from Experiment 2a replicated a DC effect between the symbolically represented ball trajectory, indicating the ball destination, and the response position in the Destination-Instructions group.

Despite instructions in the Hand-Instructions group explicitly oriented participants’ attention on the task-relevant hand stimulus, that now was visible and separated from the line representing the ball destination no spatial compatibility effect concerning the opposing player’s hand and the response was observed. This result seems to indicate that neither the instructions, nor the stimulus set suffice to divert participants’ attention from spatial information on stimulus destination when the stimulus is symmetrical and motion origin is separated from motion destination. Therefore, attention seems to be automatically oriented towards the destination of the stimulus even if other task-relevant spatial information is available, that is, even if a line of identical length, indicating motion origin (i.e., the location of the opposing player’s hand), is visible. Results concerning the Hand-Instructions group from Experiment 2a suggest that the combined impact of instructions and stimulus set somehow diminished the relevance of the ball destination, echoing results from Experiment 1b. Contrary to Experiment 1b, however, in Experiment 2a separating motion origin from motion destination led participants being not capable to shift their attentional focus from motion destination to motion origin in the Hand-Instruction group since no motion origin compatibility effect occurred [see 39 for insights on the orienting of spatial attention during action observation]. The lack of the hand-response effect in the Hand-Instruction group supports the null hypothesis as it will be revealed by a subsequent Bayesian t-test (see Additional Analysis section). This finding suggests a predominance of the effect of destination of motion over that of origin by further supporting the *common coding hypothesis*. However, considering that neither a DC effect nor a hand-response compatibility effect was observed in the Hand-Instructions group in Experiment 2a, it is likely that perceptual features play some role in modulating the occurrence of DC effects.

## Experiment 2b

Although Experiment 2a suggests that motion destination overrides motion origin, this result may stem from subjects feeling inclined to envision themselves as being a player at the bottom of the court. If so, it could be that the visual information at the bottom of the screen was more visually salient to participants. To exclude this possibility, we conducted Experiment 2b where participants were presented the same stimulus setting as in Experiment 2a but the volleyball court was flipped such that the ball trajectory was presented upward and the hitting hand was presented downward. Experiment 2b was a web-based Experiment due to the intervening pandemic circumstances.

### Method

#### Participants

Sixteen students from the University of Modena and Reggio Emilia and from the University of Bologna (10 women; mean age = 28.5 years, SD = 9.0) took part in Experiment 2b. All reported themselves as being right-handed. They were selected with the same criteria adopted for previous experiments.

#### Material and apparatus

The stimuli were the same as in Experiment 2a except that the volleyball court was flipped. A 0.4 cm width line (0.38° of visual angle), with 45-degree tilt, appeared below the ball, on the left or on the right side, indicating the hand hitting the ball; moreover a 0.4 cm height line (0.38° of visual angle), with 45 degree tilt, appeared above the ball, on the right or on the left side, indicating the trajectory of the ball (see Fig 1 bottom-right Panel). All other details were the same as in Experiment 1b.

#### Design and procedure

The design and procedure were the same as in previous experiments.

### Results

Practice trials, incorrect responses (3.20% of trials) and omissions (1.48% of trials) were excluded from the analysis on the response times (RTs). RTs higher and lower than 2 standard deviations from the participant mean were also excluded (4.86% of trials).

As in previous experiments, a mixed ANOVA with *Spatial compatibility* (compatible vs. incompatible) and *Instructions group* (destination vs. hand) was conducted on RTs. The main effect of *Spatial compatibility* was significant, *F*(1,14) = 15.69, Mse = 223.76, *p =* .001, *η*_*p*_^2^ = .528, indicating that compatible responses, i.e. responses given using a response-key ipsilateral to either the ball destination (in the Destination-Instructions group) or the opposing player’s hand (in the Hand-Instruction group) were faster (M: 372 ms SE: 8.3) than incompatible responses (M: 393 ms, SE: 9.5), i.e. responses given using a response-key contralateral to either the ball destination (in the Destination-Instructions group) or the opposing player’s hand (in the Hand-Instruction group). In addition, there was a significant two-way interaction between *Spatial compatibility* and *Instructions group*, *F*(1,14) = 5.53, Mse = 1239.25, *p =* .034, *η*_*p*_^2^ = .283. The effect of *Instructions group* failed to reach significance, F < 1. Planned comparisons aimed at comparing spatially-compatible and incompatible responses in the two instructions groups (destination, hand) revealed that for the Destination-Instructions group, compatible responses, i.e. responses given using a response-key ipsilateral to the ball destination (M: 363 ms SE: 8.9) were faster than incompatible responses (M: 396 ms, SE: 13.2), i.e. responses given using a response-key contralateral to the ball destination, resulting in a 33 ms significant DC effect, *t*(7) = 3.81, *p* = .007. On the contrary, for the Hand-Instructions group, compatible responses, i.e. responses given using a response-key ipsilateral to the opposing player’s hand (M: 381 ms SE: 14.1) were slightly faster than incompatible responses (M: 389 ms SE: 13.7) i.e. responses given using a response-key contralateral to the opposing player’s hand, resulting in a 8 ms non-significant hand-response compatibility effect, *t*(7) = 1.42, *p* = .196.

An ANOVA with the same factors was conducted on the percentage errors. No main effects or interactions were significant, F_s_ < 1.

### Discussion

Results from Experiment 2b further replicated a DC effect between the ball destination and the response position in the Destination-Instructions group.

Conversely, in the Hand-Instructions group, neither a DC effect nor a spatial compatibility effect concerning the opposing player’s hand and the response were observed. It is worth noting that there was a numerical tendency towards a compatibility effect between the opposing player’s hand and the response of 8 ms that, however, was non-significant.

Therefore, Experiment 2b, in line Experiments 1b and 2a, seems to suggest that the stimulus setting (i.e., a symmetrical line indicating both motion origin and destination in Experiment 1b; separate lines indicating motion origin upward and motion destination downward in Experiment 2a; separate lines indicating motion origin downward and motion destination upward in Experiment 2b) may exert an extra influence by weakening the relevance of the ball destination, though being unable to fully divert participants from motion destination as also suggested by Experiments 2a. This may be the reason why a compatibility effect between the opposing player’s hand and the response did not emerge in the last two experiments. This finding strengthens the idea of a predominance of the effect of destination of motion over that of origin and further supports the *common coding hypothesis*, suggesting that we represent actions in terms of their consequences.

### Additional analysis

*Bayesian t-test*. We performed a Bayesian t-test aimed at testing whether the absence of a significant difference between the Compatible and Incompatible conditions in the Hand-Instruction group for Experiments 2a and 2b can be taken as evidence in favor of the null hypothesis. Indeed, based on the procedure of null hypothesis significance testing (NHST), the null hypothesis can never be accepted, one just fails to reject it. The Bayesian t-test [40] was performed on combined data from the two experiments. We used R [41] with the "Bayes factor" library and the default Jeffreys-Zellner-Slow (JZS) prior to compare the probability of the null and the alternate hypothesis (H_o_, H_1_, respectively) relative to the absence of a significant difference between the Compatible and Incompatible conditions. We found that the Bayes factor (BF_o1_) expressing the probability of the data given H_o_ (i.e., no difference) relative to H_1_ (i.e., difference) was BF_o1_ = 4.5 [42, 43]. That is, H_o_ is 4 and a half times more likely than H_1_. This results further suggests the absence of a significant Origin compatibility effect in the Hand-instruction group.

## General discussion

The present study shows a DC effect [e.g., 21, 27] with static visual stimuli symbolically representing an object’s motion destination. This effect was obtained across four experiments, in the absence of dynamic stimuli [see 21, 27 where moving stimuli were used], and despite motion was not the consequence of a participant’s response action [see 9, 26 where participants’ responses made stimuli moving on screen]. More specifically, the effect was obtained with an asymmetrical lateralized line suggesting the ball trajectory (Experiment 1a); a symmetrical line superimposed on the central ball and indicating motion origin (upward) contralateral to motion destination (downward) (Experiment 1b); two separate lines indicating motion origin (top line) and destination (bottom line) such that participants could envision themselves as being a player at the bottom of the court (Experiment 2a); two separate lines indicating motion origin (bottom line) and destination (top line) such that participants could envision themselves as being a player at the top of the court (Experiment 2b). Overall, these results suggest that as in other SRC effects [e.g., 44–47] also the symbolically represented effects of an object’s motion can act as a stimulus source and influence responses, therefore, strengthening the hypothesis that we represent action in terms of the effects it produces [e.g., 12, 13]. It is worth noting that unlike previous studies [e.g., 22] we preferred not using arrows to study potential directional trends of symbolical motion since it is well known that the arrow produces a spatial compatibility effect for the location it points at [e.g., 22; see also 23–25]. On the contrary, the effects of simple lines lacking the geometric properties of the arrows was unexplored as far as we know.

Furthermore, it is worth highlighting that a DC effect was observed in Experiment 1a regardless of whether instructions focused participants’ attention on motion origin or destination, whereas neither a DC effect nor an origin-to-response compatibility effect was observed when instructions focused participants’ attention on motion origin in 2 out of 3 of the follow-up experiments (2a, 2b). Crucially, these results seem to speak in favor of a prominence of motion destination over origin even when both destination and origin are symbolically depicted, hence perceptually available to participants, and instructions require to respond to the origin of motion (i.e., the position of the hand hitting the ball). If as highlighted by Bosbach, Prinz, and Kerzel [48], people process object motion as a shift of position relative to the starting position, then it is plausible to assume that the starting position should gain more weight in a visual context. In fact,the predominance of the effect of destination over that of origin we observed, even when both the starting and ending positions of the stimulus were symbolically depicted as separate lines (Experiments 2a, 2b), seems to reflect different weighting mechanisms for different spatial information in a visual context (see [49] for similar results obtained by manipulating the egocentric versus allocentric action perspective). That is, attention seems to be automatically oriented towards the destination of a stimulus even when other task-relevant spatial information is available, i.e., visual cues on motion origin and instructions-driven hints on attentional focus [36].

Our findings are in line with results from a previous study conducted in our lab [50] showing that performance improved when a centrally displayed, realistic volleyball ball looked as being headed toward the ipsilateral than the contralateral response key by an opposing player’s hand. In this study stimuli were still static, thus the ball destination could only be inferred. Importantly, however, no lateralized symbolic feature, possibly indicating where the ball would end up, was used. Furthermore, contrary to the present study, there we used a picture of a volleyball ball and a picture of a real hand to create our stimuli and manipulated them through Photoshop to suggest the observer the motion direction of the ball (left, right). As it happens in a classic brick breaker videogame, where a player is able to position a horizontal bar according to the anticipated ending position of a sphere bouncing around the screen, participants were able to predict the ending position of the volleyball ball in this more ecologically valid setting. Such effects entail that motion rather than necessarily be unfolding in order to be helpful to plan and execute a response can also be helpful if just anticipated.

Taken together these findings supports the *common coding hypothesis of intention and action* [12, 13], and the broader *theory of event coding* [TEC: 14; see also 15 and 16 for an earlier version of this theory known as the *ideomotor principle*] according to which people anticipate the cognitive representation of an action consequences. In our experiments people seemed to weigh motion destination, hence the consequences of certain actions, as more relevant than motion origin. This result argues in favor of a human cognitive attitude to anticipate action effects. One exception is represented by Experiment 1b showing a main effect of Spatial compatibility, hence suggesting a origin-compatibility effect may take place in the Hand-instructions group. Such result may be due to the stimulus arrangement that in Experiment 1b represents origin and destination of motion through a diagonal, symmetrical continuous line superimposed on the central ball. Therefore, it is likely that when the line representing the hand that sets the ball in motion is in continuation with the line indicating the direction of the ball itself, the spatial information concerning the hand cannot be excluded from processing and gains more weight, probably because of its physical connection with the direction of motion, which seems to be the most relevant information in this experimental setting. Future studies may explore the scientific validity of this explanation and whether a compatibility effect with origin of motion can be obtained in specific circumstances, i.e., when the opposing player’s hand is shown alone such that no other source of perceptual information could contrast with it.

In light of these novel findings, along with previous reviewed evidence that instead focuses on participants anticipation of their own actions’ effects, future research would advance through the exploration of new paradigms, suitable for the simultaneous manipulation of expected effects of other’s actions but also of one’s own actions. Their mutual weight could be investigated through the examination of the temporal dynamics of kinematic markers, in order to detect cues that are informative not only on movement planning, but also on the subsequent refining phase [i.e., online control: 51, 52]. This would allow for an in-depth analysis of the re-coding of responses in terms of distal effects of the other’s vs. the agent’s anticipated action.

Overall, the current study provides evidence in favor of the independence of DC effects from other SRC effects [e.g., 53].

## Supporting information

S1 File Contains the alternative analyses where spatial compatibility was computed between the ball destination and the response location in both Instructions groups. (DOC)

## References

[1] Fitts PM, Seeger CM. SR compatibility: spatial characteristics of stimulus and response codes. J Exp Psychol. 1953;46(3): 199–210. doi: 10.1037/h0062827 13084867

[2] Fitts PM, Deininger RL. SR compatibility: correspondence among paired elements within stimulus and response codes. J Exp Psychol. 1954;48(6): 483–492. doi: 10.1037/h0054967 13221745

[3] Kornblum S, Lee JW. Stimulus-response compatibility with relevant and irrelevant stimulus dimensions that do and do not overlap with the response. J Exp Psychol Hum Percept Perform. 1995;21(4): 855–875. doi: 10.1037//0096-1523.21.4.855 7643052

[4] Proctor RW, Vu KPL. Stimulus-response compatibility principles: Data, theory, and application. CRC press; 2006.

[5] Hommel BE, Prinz WE. Theoretical issues in stimulus-response compatibility. Elsevier Science/JAI Press; 1997.

[6] Simon JR. Reactions toward the source of stimulation. J Exp Psychol. 1969;81: 174–176. doi: 10.1037/h0027448 5812172

[7] Simon JR. The effects of an irrelevant directional cue on human information processing. In: Proctor RW, Reeve TG editors. Stimulus-response compatibility: An integrated perspective. Amsterdam: North-Holland; 1990. pp. 31–86.

[8] Hommel B. Inverting the Simon effect by intention. Psychol Res. 1993;55(4): 270–279.

[9] Ansorge U. Spatial intention–response compatibility. Acta Psychol. 2002;109(3): 285–299. doi: 10.1016/s0001-6918(01)00062-2 11881904

[10] Kunde W. Response-effect compatibility in manual choice reaction tasks. J Exp Psychol Hum Percept Perform. 2001;27(2): 387–394. doi: 10.1037//0096-1523.27.2.387 11318054

[11] Scerrati E, D’Ascenzo S, Lugli L, Iani C, Rubichi S, Nicoletti R. Exploring the Role of Action Consequences in the Handle-Response Compatibility Effect. Front. Hum. Neurosci. 2020;14: 286. doi: 10.3389/fnhum.2020.00286 Available from: https://www.frontiersin.org/articles/10.3389/fnhum.2020.00286/full 32848666 PMC7411217

[12] Prinz W. A common coding approach to perception and action. In: Neumann O, Prinz W, editors. Relationships between perception and action: Current Approaches. Berlin: Springer; 1990. pp. 167–201.

[13] Prinz W. Perception and action planning. Eur J Cogn Psychol. 1997;9(2): 129–154.

[14] Hommel B, Müsseler J, Aschersleben G, Prinz W. The theory of event coding (TEC): A framework for perception and action planning. Behav Brain Sci. 2001;24(5): 849–878. doi: 10.1017/s0140525x01000103 12239891

[15] Greenwald AG. Sensory feedback mechanisms in performance control: with special reference to the ideo-motor mechanism. Psychol Rev. 1970;77(2): 73–99. doi: 10.1037/h0028689 5454129

[16] James W. The principles of psychology. Read Books Ltd; 2013.

[17] Elsner B, Hommel B. Effect anticipation and action control. J Exp Psychol Hum Percept Perform. 2001;27(1): 229–240. doi: 10.1037//0096-1523.27.1.229 11248937

[18] Jeannerod M. The 25th Bartlett Lecture: To act or not to act: Perspectives on the representation of actions. Q J Exp Psychol. 1999;52(1): 1–29. 10.1080/713755803 10101973

[19] Pfister R, Pfeuffer C.U, Kunde W. Perceiving by proxy: Effect-based action control with unperceivable effects. Cognition. 2014;132(3): 251–261. doi: 10.1016/j.cognition.2014.04.012 24853628

[20] Shin YK, Proctor RW, Capaldi EJ. A review of contemporary ideomotor theory. Psychol Bull. 2010;136(6): 943–974. doi: 10.1037/a0020541 20822210

[21] Michaels CF. SR compatibility between response position and destination of apparent motion: Evidence of the detection of affordances. J Exp Psychol Hum Percept Perform. 1988;14(2): 231–240. doi: 10.1037//0096-1523.14.2.231 2967878

[22] Proctor RW, Van zandt T, Lu CH, Weeks DJ. Stimulus-response compatibility for moving stimuli: Perception of affordances or directional coding? J Exp Psychol Hum Percept Perform. 1993;19(1): 81–91. 8440990 10.1037//0096-1523.19.1.81

[23] Bashore T. R. Stimulus-response compatibility viewed from a cognitive psychophysiological perspective. In Proctor R. W. & Reeve T. G. (Eds.), Stimulus-response compatibility: An integrated perspective. Amsterdam: New-Holland; 1990. pp. 183–223.

[24] Weeks D. J., & Proctor R. W. Compatibility effects for orthogonal stimulus-response dimensions. Journal of Experimental Psychology: General. 1990; 119: 355–366.

[25] Pellicano A., Lugli L., Baroni G., & Nicoletti R. The Simon effect with conventional signals: A time-course analysis. Experimental Psychology. 2009: 56(4): 219–227. doi: 10.1027/1618-3169.56.4.219 19439393

[26] Tagliabue M, Falciati L, Umiltà C, Massaccesi S. Compatibilità spaziale stimolo-meta e compatibilità spaziale meta-risposta in situazioni ecologiche. Giornale Italiano di Psicologia. 2006;33(1): 143–174.

[27] Classen C, Kibele A. Action induction due to visual perception of linear motion in depth. Psychol Res. 2017;81(1): 131–142. doi: 10.1007/s00426-015-0724-3 26586291

[28] D’Ascenzo S, Scerrati E, Villani C, Galatolo R, Lugli L, Nicoletti R. Does Social distancing affect the processing of brand logos?. Brain and Behavior. 2022 doi: 10.1002/brb3.2501 35212187 PMC8933757

[29] Marzola G, D’Ascenzo S, Lugli L. L’associazione spaziale dei simboli matematici “meno e più”. Giornale Italiano di Psicologia. In press.

[30] Scerrati E., Rubichi S., Nicoletti R., & Iani C. (2022). Emotions in motion: affective valence can influence compatibility effects with graspable objects. *Psychological Research*, 1–12. doi: 10.1007/s00426-022-01688-6 35616712

[31] Cohen J. The statistical power of abnormal-social psychological research: a review. *The Journal of Abnormal and Social Psychology*. 1962;65(3), 145–153. doi: 10.1037/h0045186 13880271

[32] Sedlmeier P., & Gigerenzer G. Do studies of statistical power have an effect on the power of studies? *Psychological Bulletin*. 1989;105(2), 309–316. 10.1037/0033-2909.105.2.309.

[33] Dienes Z. Using Bayes to get the most out of non-significant results. *Front*. *Psychol*. 2014;5:781. doi: 10.3389/fpsyg.2014.00781 25120503 PMC4114196

[34] Faul F, Erdfelder E, Lang AG, Buchner A. G*Power 3: A flexible statistical power analysis program for the social, behavioral, and biomedical sciences. Behav Res Methods. 2007;39(0): 175–191. doi: 10.3758/bf03193146 17695343

[35] Cohen J. Statistical power analysis for the behavioral sciences. Hillsdale, NJ: Erlbaum; 1988.

[36] Loftus GR, Masson ME. Using confidence intervals in within-subject designs. Psychon Bull Rev. 1994;1(4): 476–490. doi: 10.3758/BF03210951 24203555

[37] Anwyl-Irvine A. L., Massonnié J., Flitton A., Kirkham N., & Evershed J. K. Gorilla in our midst: An online behavioral experiment builder. *Behavior research methods*. 2020; 52(1): 388–407. doi: 10.3758/s13428-019-01237-x 31016684 PMC7005094

[38] Scerrati E., Marzola G., Villani C., Lugli L., D’Ascenzo S. Nuovi scenari per gli esperimenti in psicologia: la modalità online. Giornale Italiano di Psicologia. In press.

[39] Betti S, Castiello U, Guerra S, Sartori L. Overt orienting of spatial attention and corticospinal excitability during action observation are unrelated. PLoS One. 2017;12(3), e0173114. doi: 10.1371/journal.pone.0173114 28319191 PMC5358745

[40] Rouder J N, Speckman P L, Sun D, Morey R D, & Iverson G. Bayesian t tests for accepting and rejecting the null hypothesis. Psychonomic bulletin & review. 2009; 16(2): 225–237. doi: 10.3758/PBR.16.2.225 19293088

[41] R Core Team. R: A language and environment for statistical computing. R Foundation for Statistical Computing. Vienna: Austria Retrieved from, https://www.R-project.org/ 2016.

[42] Wagenmakers E. J. A practical solution to the pervasive problems of p values. Psychon. Bull. Rev. 2007;14: 779–804. doi: 10.3758/bf03194105 18087943

[43] Raftery A. E. Bayes factors and BIC. Sociol. Methods. Res. 1999; 27: 411–427.

[44] Scerrati E, Lugli L, Nicoletti R, & Umiltà C. Comparing Stroop-like and Simon effects on perceptual features. Scientific reports 2017; 7(1): 1–11. 29259275 10.1038/s41598-017-18185-1 PMC5736580

[45] D’Ascenzo S, Lugli L, Nicoletti R, & Umiltà C. Practice effects vs. transfer effects in the Simon task. Psychological Research. 2021; 85(5): 1955–1969. doi: 10.1007/s00426-020-01386-1 32770264 PMC8289792

[46] Pellicano A, Lugli L, Binkofski F, Rubichi S, Iani C, Nicoletti R. The unimanual handle-to-hand correspondence effect: evidence for a location coding account. Psychological Research. 2019; 83: 1383–1399. doi: 10.1007/s00426-018-1009-4 29651534

[47] D’Ascenzo S, Lugli L, Baroni G, Guidotti R, Rubichi S, Iani C, et al. Visual versus auditory Simon effect: A behavioural and physiological investigation. Quarterly Journal of Experimental Psychology. 2018; 71: 917–930. doi: 10.1080/17470218.2017.1307429 28293982

[48] Bosbach S, Prinz W, Kerzel D. A Simon effect with stationary moving stimuli. J Exp Psychol Hum Percept Perform. 2004;30(1): 39–55. doi: 10.1037/0096-1523.30.1.39 14769067

[49] Scerrati E, Iani C, Lugli L, Nicoletti R, & Rubichi S. Do my hands prime your hands? The hand-to-response correspondence effect. Acta Psychologica. 2020; 203, 103012. doi: 10.1016/j.actpsy.2020.103012 31981827

[50] Scerrati E, Lugli L, Scorolli C. Quando immaginare gli effetti di un’azione genera compatibilità spaziale. Uno studio preliminare [When imagining the effects of an action generates spatial compatibility: An introductory study]. *Giornale Italiano di Psicologia*. 2020;3(4): 981–989.

[51] Glover S. Separate visual representations in the planning and control of action. Behav Brain Sci. 2004;27(1): 3–24. doi: 10.1017/s0140525x04000020 15481943

[52] Scorolli C, Pellicano A, Nicoletti R, Rubichi S, Castiello U. The Simon effect in action: Planning and/or on‐line control effects? Cogn Sci. 2015;39(5): 972–991. doi: 10.1111/cogs.12188 25330713

[53] Styrkowiec P. Space and motion stimulus-response correspondence (SRC) effects in a single task: Evidence for distinct SRC phenomena. Exp Psychol. 2016;63(5): 297–306. 27832733 10.1027/1618-3169/a000335

