# Supplementary material for: Compatibility effects with destination and origin of motion
Source: PLoS One. 2023 Feb 17;18(2):e0281829. doi: 10.1371/journal.pone.0281829 (PMC9937485; doi:10.1371/journal.pone.0281829)
Supplement: S1 File — (DOC) [file pone.0281829.s001.doc]

**Alternative Analyses**

For further insights on the results from the four experiments, we also conducted a MIXED ANOVA where spatial compatibility was computed between the ball destination and the response location in both Instructions groups. A destination-compatibility (DC) effect, therefore, rests on faster compatible than incompatible responses to the destination of the ball, whereas a origin-compatibility effect rests on faster incompatible than compatible responses to the destination of the ball given that destination and origin of motion are always presented contralateral to each other in the current experiments.

**Experiment 1a.** The main effect of *Spatial compatibility* was significant, *F*(1,21) = 109.64, Mse = 132.37, *p <* .001, *η_p_*^2^ =.839, indicating that compatible responses, i.e. responses given using a response-key ipsilateral to the ball destination (M: 321 ms SE: 5.2) were faster than incompatible responses (M: 357 ms, SE: 6.1), i.e. responses given using a response-key contralateral to the ball destination. Planned comparisons were conducted on the magnitude of the DC effect (mean response time on incompatible trials minus mean response time on compatible trials), separately for each group. There was a significant DC effect of 38 ms in the Destination-instruction group, *t*(11) = 7.17, *p* < .001, and a significant DC effect of 32 ms in the Hand-instruction group, *t*(10) = 8.16, *p* < .001. All other effects failed to reach significance, F_s_ < 1.

An ANOVA with the same factors was conducted on the percentage errors (PE). The main effect of *Spatial compatibility* was significant, *F*(1,21) = 11.06, Mse = 7.67, *p =* .003, *η_p_*^2^ =.345, indicating that compatible responses (M: 2.4%, SE: 0.4) were more accurate than incompatible responses (M: 5.1%, SE: 0.9). Planned comparisons were conducted on the magnitude of the DC effect (mean percentage error on incompatible trials minus mean percentage error on compatible trials), separately for each group. There was a non-significant DC effect of 2.2 % in the Destination-instruction group, *t*(11) = 1.80, *p* < .099 and a significant DC effect of 3.2 % in the Hand-instruction group, *t*(10) = 3.10, *p* < .011.

All other effects failed to reach significance, F_s_ < 1.

**Experiment 1b.** The main effect of *Spatial compatibility* was not significant, *F* < 1. Likewise, the main effect of *Instructions group* did not reach statistical significance*,* F(1,22) = 3.28, p = .084. Crucially, there was a significant two-way interaction between *Spatial compatibility* and *Instructions group*, *F*(1,22) = 6.01, Mse = 154.81, *p =* .023, *η_p_*^2^ =.215. Planned comparisons aimed at comparing spatially-compatible and incompatible responses in the two instructions groups (destination, hand) revealed that for the Destination-Instructions group, compatible responses, i.e. responses given using a response-key ipsilateral to the ball destination (M: 403 ms SE: 7.1) were faster than incompatible responses (M: 412 ms, SE: 6.5), i.e. responses given using a response-key contralateral to the ball destination, resulting in a 9 ms significant DC effect, *t*(11) = 2.35, *p* = .038. On the contrary, for the Hand-Instructions group, there were slightly faster incompatible (M: 381 ms SE: 10.6) than compatible (M: 390 ms SE: 10.2) destination and responses. This difference, however, did not reach statistical significance, *t*(11) = 1.39, *p* = .189.

A mixed ANOVA with the same factors was conducted on the percentage errors. Both the main effect of *Spatial compatibility* and of *Instructions group* were not significant, both *F* < 1. Crucially, there was a significant two-way interaction between *Spatial compatibility* and *Instructions group*, *F*(1,22) = 8.72, Mse = 8.59, *p =* .007, *η_p_*^2^ =.284. Planned comparisons aimed at comparing spatially-compatible and incompatible responses in the two instructions groups (destination, hand) revealed that for the Destination-Instructions group, compatible responses (M: 3.6 % SE: 1.1) were more accurate than incompatible responses (M: 6.3 %, SE: 1.5), resulting in a significant DC effect of 2.7%, *t*(11) = 2.69, *p* = .021. On the contrary, for the Hand-Instructions group, there were slightly more accurate incompatible (M: 5.1 %, SE: 1.2) than compatible (M: 7.4 % SE: 2.4) destination and responses. This difference, however, did not reach statistical significance, *t*(11) = 1.69, *p* = .118.

**Experiment 2a.** Results showed a main effect of *Spatial compatibility, F*(1,30) = 7.58, Mse = 301.00, *p =* .01, *η_p_*^2^ =.202, indicating that compatible responses, i.e. responses given using a response-key ipsilateral to the ball destination (M: 346 ms SE: 5.3) were faster than incompatible responses (M: 358 ms, SE: 5.9), i.e. responses given using a response-key contralateral to the ball destination . Pairwise comparisons were conducted on the magnitude of the DC effect (mean response time on incompatible trials minus mean response time on compatible trials), separately for each group. There was a significant DC effect of 18 ms in the Destination-instruction group, *t*(15) = 2.68 , *p* = 0.17, whereas there was a non-significant DC effect of 6 ms in the Hand-instruction group, *t*(15) = 1.04 , *p* = .311. All other effects failed to reach significance, *F_s_* < 2.10, *p_s_* > .157.

An ANOVA with the same factors was conducted on the percentage errors. The main effect of spatial compatibility failed to reach significance, *F* = 3.36, p = .077. All other effects were non significant, *F_s_* < 1.

**Experiment 2b.** Results showed a main effect of *Spatial compatibility, F*(1,14) = 5.53, Mse = 223.76, *p =* .03, *η_p_*^2^ =.283, indicating that compatible responses, i.e. responses given using a response-key ipsilateral to the ball destination (M: 376 ms SE: 8.2) were faster than incompatible responses (M: 388 ms, SE: 9.6), i.e. responses given using a response-key contralateral to the ball destination. In addition, there was a significant interaction between *Spatial compatibility* (compatible vs. incompatible) and *Instructions group* (destination vs. hand), *F*(1,14) = 15.69, Mse = 3511.34, *p =* .001, *η_p_*^2^ =.528. Planned comparisons aimed at comparing spatially-compatible and incompatible responses in the two instructions groups (destination, hand) revealed that for the Destination-Instructions group, compatible responses, i.e. responses given using a response-key ipsilateral to the ball destination (M: 363 ms SE: 11.6) were faster than incompatible responses (M: 396 ms, SE: 13.6), i.e. responses given using a response-key contralateral to the ball destination, resulting in a significant DC effect of 33 ms, *t*(7) = 3.81, *p* =.007. On the contrary, for the Hand-Instructions group, there were slightly faster incompatible (M: 381 ms SE: 13.6) than compatible (M: 389 ms SE: 11.6) responses. This difference, however, did not reach statistical significance, *t*(7) = 1.42, *p* =.196. No other effects were significant, *F* < 1.

An ANOVA with the same factors was conducted on the percentage errors. No main effects or interactions were significant, F_s_ < 1.
